# Potential Applications of Extracellular Vesicles in Solid Organ Transplantation

**DOI:** 10.3390/cells9020369

**Published:** 2020-02-05

**Authors:** Cristina Grange, Linda Bellucci, Benedetta Bussolati, Andrea Ranghino

**Affiliations:** 1 Department of Medical Sciences, University of Turin, 10126 Turin, Italy; cristina.grange@unito.it (C.G.); andrea.ranghino@unito.it (A.R.); 2 Department of Molecular Biotechnology and Health Sciences, University of Turin; 10126 Turin, Italy; linda.bellucci@unito.it; 3 SOD Nefrologia, Dialisi e Trapianto Rene, AOU Ospedali Riuniti, 60126 Ancona, Italy

**Keywords:** exosomes, regenerative medicine, machinery perfusion, transplant, preconditioning

## Abstract

Extracellular vesicles (EVs) play an important role in cell-to-cell communication by delivering coding and non-coding RNA species and proteins to target cells. Recently, the therapeutic potential of EVs has been shown to extend to the field of solid organ transplantations. Mesenchymal stromal cell-derived EVs (MSC-EVs) in particular have been proposed as a new tool to improve graft survival, thanks to the modulation of tolerance toward the graft, and to their anti-fibrotic and pro-angiogenic effects. Moreover, MSC-EVs may reduce ischemia reperfusion injury, improving the recovery from acute damage. In addition, EVs currently considered helpful tools for preserving donor organs when administered before transplant in the context of hypothermic or normothermic perfusion machines. The addition of EVs to the perfusion solution, recently proposed for kidney, lung, and liver grafts, resulted in the amelioration of donor organ viability and functionality. EVs may therefore be of therapeutic interest in different aspects of the transplantation process for increasing the number of available organs and improving their long-term survival.

## 1. Introduction

Solid organ transplantation represents the gold standard treatment for patients with end-stage organ failure. Specifically, kidney transplantation has become a routine procedure because of its beneficial effects on patient survival and quality of life, together with its economic aspects [1]. Although the global observatory on donation and transplantation reported a total of 139,024 solid organ transplants worldwide with 90,306 kidneys in 2017, this met less than 10% of the global need [2]. Data from Eurotransplant [3], NHS-UK [4], and US registries [5] show that 141,568 patients are waiting for a transplant, 82% of which are kidney transplants. Therefore, the gap existing between the need for transplants and organ availability represents a major challenge to be addressed by scientific community [6]. To reduce this gap, novel strategies have to be explored. The main option being explored at present is the increase of the pool of deceased donors, including donors after circulatory death (DCDs), which actually represent about 20% of the deceased donors worldwide, and older donors with comorbidities such as hypertension, mild renal impairment, and death from cerebrovascular events (extended criteria donors, ECDs) [7]. Nevertheless, organs from DCDs and ECDs are more prone to developing an ischemic-reperfusion injury (IRI) compared to standard donors, and consequently represent an increased risk of primary non-function and delayed graft function (DGF) [8]. In addition, long-term graft survival is still a critical factor that needs to be improved.

Among the different strategies in regenerative medicine, EVs have been recently recognized as a promising and innovative tool with which to accelerate tissue recovery after organ damage. EVs are a heterogeneous group of membranous vesicles that possess a central role in the mechanisms of cell-to-cell communication [9,10]. In the last decade, interest and knowledge in the field of EVs has increased enormously, and it is now well established that EVs may influence the function of target cells by transferring bioactive molecules and genetic materials, inducing epigenetic changes in recipient cells [11,12,13].

**Figure 1:**
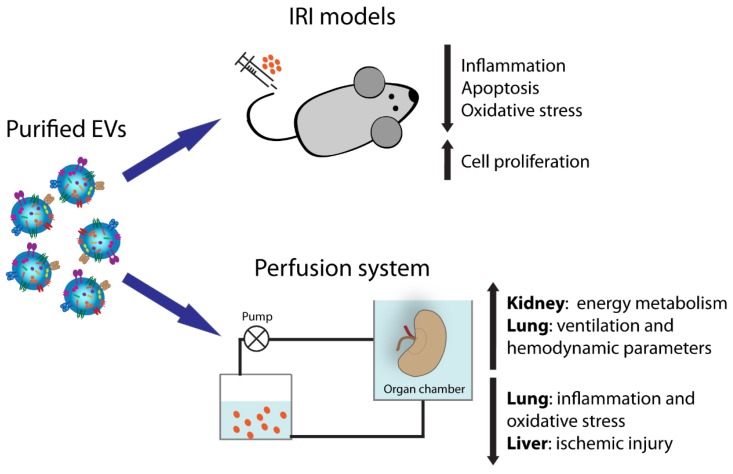
Extracellular vesicle (EV) activities in solid organ transplantation.

In this review, we present the current literature regarding the potential application of stem-cell-derived EVs, dissecting their possible application as an innovative therapeutic tool to precondition grafts before transplant as well as to prevent ischemic/reperfusion damage (Figure 1). In particular, we describe their use in pre-transplant solid organ preservation in association with normothermic and hypothermic perfusion machines. In addition, their role in the limitation of IRI is highlighted for kidney, liver, lung, and heart. Finally, we present their immunomodulatory properties in bone marrow transplantation.

## 2. Stem-Cell-Derived EVs and Regenerative Medicine

EVs released by healthy cells are very heterogeneous in size and composition, and they can be classified based on their origin and dimension into two main categories: small EVs, ranging between 30 and 100 nm, and large EVs, ranging between 50 and 1000 nm [14].

Among small EVs, exosomes are the most characterized vesicles, considered to originate from multivesicular bodies after their fusion with the cell membrane [15]. However, other subtypes of small EVs different from the multivesicular-body-derived exosomes have been identified, for instance after plasma membrane budding [14].

Large EVs, also called microvesicles/ectosomes, comprise different populations of vesicles originating from the budding of the plasma membrane [16]. The different EV populations express common and specific surface markers. For instance, tetraspanins such as CD9, CD81, and CD63 are mainly expressed by small EVs [14]. In addition, small EVs are characterized by the presence of molecules of the endosomal sorting complex required for transport (ESCRT), heat shock proteins (HSP70 and HSP90), and auxiliary proteins (ALIX, TSG101, and VPS4). In terms of variance, large EVs are specifically characterized by expression of the CD40 ligand [17,18]. The detailed composition of EV cargo has been deeply dissected and several databases collecting these results are now available, such as EVpedia [19], Exocarta [20], and Vesiclepedia [21]. EVs can be isolated from the majority of body fluids such as plasma and serum, amniotic and seminal fluids, saliva, urine, or nasal and bronchial lavage fluids [9,22].

Is important to take into consideration that a limitation to consistent EV characterization is the variability in EV isolation protocols. Depending on the size of EVs and on the fluids of origin, different techniques can be utilized, including ultra-high-speed centrifugation, polymer precipitation, immunoaffinity capture, or microfluidics-based techniques, among others [23]. Rigor criteria for EV isolation and characterization were recently proposed by the International Society for Extracellular Vesicles (ISEV) [14].

Stem-cell-derived EVs possess many characteristics in common with the originating cells; for instance, they carry some transcription factors classically expressed by stem cells, such as Nanog and Oct-4, as well as stem (CD133 and c-Kit) and mesenchymal markers (CD105, CD29, and CD73) [24]. It has been clearly demonstrated that stem-cell-derived EVs recapitulate the pro-regenerative capacity of the cells of origin and, in particular, those derived from mesenchymal stromal cells (MSCs) appear the ideal candidates to favor tissue regeneration. MSC-EVs may be isolated from MSCs derived from different adult tissues such as bone marrow, peripheral and cord blood, adipose tissue, or neonatal birth-associated tissues including placenta and umbilical cord [25]. Several studies have shown that MSC-EVs possess strong pro-regenerative properties using preclinical models of renal, lung, liver, and heart injuries, mimicking the beneficial effect of the cells themselves [14,26,27]. The activity of EVs mainly results in the reduction of apoptosis, oxidative stress, and inflammation and in increase of cell proliferation [24,28,29].

## 3. Normothermic and Hypothermic Perfusion Machines

In order to increase the number of successful transplants, the use of machine perfusion is currently proposed to ameliorate the function of organs from marginal donors such as DCDs and ECDs. Dynamic perfusion of organs appears a useful strategy to evaluate pretransplant graft function, limiting the discard rate [30,31,32]. Moreover, this approach reduces the incidence of DGF in recipients receiving organs from ECDs and DCDs.

At present, dynamic machine perfusion can be done in hypothermic (HMP) or in normothermic (NMP) conditions with or without oxygen. Several studies have demonstrated that both HPM and NPM are useful in the assessment of organ viability prior to transplantation [32,33,34]. Specifically, HMP is able to reduce DGF and to increase the graft survival of organs harvested from ECDs, but conflicting results have been reported on the beneficial effects of HMP on grafts from DCDs [35,36,37,38,39,40]. Another beneficial effect of HMP is the removal of inflammatory mediators that may have detrimental effects on graft function. The delivery of oxygen added to the hypothermic perfusate may help to restore adenosine triphosphate (ATP) content [41,42,43,44]. Because of the unknown effects of this oxygenated perfusion on transplanted patients, a large international randomized controlled trial has been planned to investigate the beneficial effects of oxygenated short-term perfusion of kidneys from ECDs (Consortium for Organ Preservation in Europe COPE Trials) [45].

As oxygenated machine perfusion, NMP may protect organs from IRI by restoring ATP levels [46,47]. In particular, ex vivo normothermic perfusion, consisting of circulation through the harvested organs of warm oxygenated red-cell-based solution, is able to restore the metabolism and function of the graft prior to transplantation [48,49,50]. NMP could offer a better evaluation of organ viability compared to HMP, especially in kidney and liver grafts because of urine or bile production, together with a better preservation of graft function [51].

Both HMP and NMP allow the delivery of targeted therapies to organs prior to transplantation. In particular, these approaches offer the potential to explore the effects of several therapeutic strategies, such as gene-silencing, nanoparticles, and cell therapies, in a fully functioning graft [52,53,54,55,56,57].

## 4. EVs for Kidney Transplant

An innovative EV-based application for organ preservation is the use of EVs in the perfusion solution. A first report in the literature recently demonstrated that EVs released by MSCs, delivered in the perfusate during organ cold perfusion (4 h), preserve and protect kidney function. Histological and genetic analyses on EV-treated kidneys revealed upregulation of enzymes involved in energy metabolism and reduction of global ischemic damage. In addition, the analysis of lactate, LDH, and glucose in the effluent fluid confirmed a greater use of energy substrates by EV-treated kidneys, supporting the report of improved functionality (Table 1) [58].

Moreover, an extensive number of publications have highlighted the beneficial effect of EVs in preclinical models of IRI, further implying their possible application to limit organ damage [9]. In particular, EVs isolated from different MSC sources [59,60,61,62] have been shown to accelerate renal recovery after damage, promoting cell proliferation and blocking inflammation and apoptosis when intravenously injected after IR damage [63]. The mechanisms of action reported appear different between the EV sources: MSC-EVs obtained from Wharton’s jelly stimulate tubular proliferation and reduce inflammation and apoptosis via mitochondrial protection [61,62], while those from cord blood promote tubular dedifferentiation and proliferation by the transfer of human HGF [60]. Moreover, EVs isolated from bone marrow MSCs were protective mainly by suppressing inflammation when injected under the renal capsule [64]. In addition, EVs obtained from MSCs isolated from glomeruli have also been demonstrated to be capable of reducing ischemic damage [65].

Moreover, a recent publication demonstrated that EVs isolated from the venal perfusate of rats subjected to remote ischemia preconditioning ameliorated renal function when injected into another animal with IRI. To explore the underlying mechanism, authors tested in vivo, in the same IRI model, the effect of EVs released by human proximal tubular cells cultured in hypoxia, supporting the thesis that remote ischemia precondition activates a repairing program into tubular cells by the release of pro-regenerative EVs [66].

Whereas all the studies mentioned above evaluated classical ischemic damage in models of renal artery clamping, Wu and co-workers tested for the first time the effect of EVs in a rat model of IRI after DCD renal transplantation [67]. The authors confirmed that Wharton’s jelly MSC-EVs, intravenously injected after renal transplantation, mitigated renal damage, improving survival and function. In particular, MSC-EVs were shown to reduce cell apoptosis and inflammation, to stimulate HGF production, and subsequently to alleviate fibrosis [67].

## 5. EVs for Lung Transplantation

Adult lung transplantation is considered the most effective strategy for end-stage pulmonary disease, although the reported 5-year survival rate is only 50% [73]. Infections, immunomodulation, and IRI are in fact some of the aspects involved in lung transplant failure [74]. Through ex vivo lung perfusion, donor lungs can be evaluated and reconditioned, while organs are perfused and ventilated [75]. The use of MSC-EVs has been proposed as a valid alternative for the rehabilitation of marginal human lungs [68]. Upon administering MSC-EVs in the perfusion fluid, a dose-dependent increase of alveolar fluid clearance, a decrease of lung weight gain, and an improvement of airway and hemodynamic parameters were observed as compared to perfusion alone (Table 2). Moreover, the study showed that CD44 was involved in the EV uptake mechanism, as the efficacy of MSC-EVs decreased with the administration of anti-CD44 antibody.

A significant improvement of inflammatory conditions has also been ascribed to the EV effect on lung bacterial infections. For example, MSC-EVs have been demonstrated to be effective in restoring lung protein permeability and reducing inflammation in *Escherichia-coli*-endotoxin-induced acute lung injury in mice. In particular, MSC-EV treatment restored protein permeability and reduced inflammation, extravascular lung water, and total protein levels in the bronchoalveolar lavage fluid, demonstrating a reduction in pulmonary edema [76]. On this path, in a recent work, the effects of MSC-EVs were investigated in an ex vivo perfused human lung model, injured with severe *E. coli* pneumonia [69]. The paper confirmed a significant increase of alveolar fluid clearance and decrease in protein permeability, as well as the lowering of the bacterial load and the neutrophil count in the injured alveolus (Table 2). MSC pretreatment with a toll-like-receptor 3 agonist before the isolation of EVs increased their bactericidal activity.

Moreover, Stone and colleagues demonstrated the attenuation of IR dysfunction in lungs after treatment with MSC-EVs both in vivo and in ex vivo perfusion systems [70]. In particular, they observed a decrease of pro-inflammatory cytokines and upregulation of keratinocyte growth factor, PGE2, and IL-10. Recently, in a mouse model of ex vivo lung perfusion, EV-treated organs showed decreased vascular resistance and a rise of perfusate nitric oxide metabolites. Moreover, EV treatment prevented the reduction in pulmonary ATP and increased the medium–high-molecular-weight hyaluronan in the perfusate. The genes modulated in the pulmonary tissue by EV administration were involved in anti-inflammatory and anti-oxidative stress pathways [71].

## 6. EVs for Liver Transplantation

The use of EVs released by stem cells as an innovative option to improve the viability of pre-transplant livers was recently assessed in a model of ex vivo rat liver NMP. HLSC-EVs (EVs isolated from human liver stem cells) were added to perfusate 15 min after the initiation of NMP and administered for 4 h within the perfusate. The results showed that HLSC-EVs limited the progression of ischemic injury, with a significant reduction of the levels of aspartate aminotransferase and alanine aminotransferase and a decrease of histological damage compared with results of NMP alone (Table 2) [72]. Moreover, the authors demonstrated that HLSC-EVs were uptaken by hepatocytes, supporting the thesis that EVs may recondition liver cells before transplantation [72].

Moreover, the potential therapeutic use of stem-cell-derived-EVs for liver regeneration, has been also clearly demonstrated in pre-clinical models of liver IRI. In fact, hepatic ischemia and related inflammation should be limited to avoid complication after liver transplantation [77]. The intravenous injection of murine MSC-EVs prior to IRI reduced the area of necrosis and apoptosis with concomitant increased liver function [77]. In addition, MSC-EVs have been shown to limit liver inflammation and oxidative stress [77]. Similar results were obtained using EVs isolated from MSCs from inducible pluripotent stem cells [78] or bone marrow [79]. Recently, Yao et al. demonstrated that human umbilical cord MSC-EVs protect hepatic apoptosis post-IRI, modulating neutrophils and reducing oxidative stress [80].

## 7. Stem-Cell-Derived EVs as Future Therapeutics in Heart Transplantation

EVs have been shown to be powerful allies against cardiovascular damage. Some important interconnected effects related to EVs could improve the success of a heart transplantation, including immunomodulatory properties, the improvement of heart function and vessel formation, and the amelioration of myocardial function during IRI [81].

Much evidence confirms the hypothesis that cardiac progenitor cells release pro-regenerative and anti-fibrotic EVs in response to hypoxic conditions [82,83], mainly due to their miRNA cargo [82]. Moreover, cardiac-progenitor-cell-derived EVs, released into their environment, can stimulate migration of endothelial cells [84] and inhibit both cardiac fibroblast activation and collagen synthesis [85].

In parallel, MSC-EV treatment has also been proven as a therapeutic option to limit ischemic damage in the heart. In particular, MSC-EV administration increased phosphorylated-Akt and phosphorylated-GSK-3β, as well as ATP/NADH level, and could reduce phosphorylated-c-JNK and inflammatory response in ischemic/reperfused hearts [86].

## 8. EVs for Islet Transplantation

Today, there are still many factors that limit the success of pancreatic islet transplantation, including islet source limitation, sub-optimal engraftment, lack of oxygen and blood supply for transplanted islets, and immune rejection [87]. In parallel with the other described organs, MSC-EVs may also be of benefit for islet transplantation.

One of the primary reasons for apoptosis and reduced beta-cell function in transplants is hypoxic damage. Recently, EVs from human-umbilical-cord-derived MSCs were shown to have a therapeutic effect on the survival and function of neonatal porcine islets exposed to hypoxia [88]. The use of EVs, in comparison with medium alone, enhanced the yield and survival of porcine islets, and showed an improvement of the function through the amelioration of mitochondrial respiration efficiency [88].

In addition, Di Wen and colleagues showed that MSC-EV administration through delivery of small RNAs promoted islet function and inhibited immune rejection [89]. In a mouse model, they used MSC-EVs transfected with shFas and anti-miR-375 in order to silence Fas and miR-375 in human islets, observing an improvement of islet viability and function. Moreover, the authors observed the inhibition of peripheral blood mononuclear cell proliferation and the enhancement of T-cell regulatory function. Based on these works, EVs from different sources appear of interest to increase the possibility of successful islet transplantation.

## 9. Role of MSC-EVs in the Amelioration of Graft Versus Host Disease

EVs derived from bone marrow MSCs possess an immunosuppressive potential that can be harnessed to treat graft versus host disease (GVHD), which today represents the greatest complication after allogeneic transplantation. The majority of the literature on the subject has generically focused on the effects of the whole MSC secretome, including EVs and soluble factors. Recently, the specific role of EVs has been highlighted, showing an effect on innate and adaptive immunity (Table 2).

For example, in 2005, Aggarwal and Pittenger highlighted that the secretome, released by MSCs, be responsible for modulation of immune reaction, involved in GVHD [90]. In fact, if co-cultured with purified subpopulations of immune cells, human MSCs were able to switch an inflammatory response into a tolerant phenotype. In particular, MSCs induced mature dendritic cells type 1 and type 2 to decrease TNF-α and to increase IL-10 secretion, respectively; they also induced T helper 1 lymphocytes and natural killer cells to decrease interferon (IFN) γ secretion. In addition, they enhanced a regulatory response, causing the T helper 2 cells to increase secretion of IL-4, increasing the proportion of regulatory T cells and producing prostaglandin (PG) E2 [90].

Moreover, soluble factors released by MSCs, such as vascular endothelial growth factor and IL-6, were shown to inhibit T-cell proliferation and to be involved in a partial inhibition of dendritic cell differentiation [91]. Selmani et al. not only confirmed the role of the MSC secretome in modulating innate immunity, but they also sustained its strong modulation of adaptive immunity [92]. Moreover, they reported that the nonclassic HLA class I molecule HLA-G is responsible for the immunomodulatory properties of MSCs [92].

In a recent work, it was shown that bone marrow MSC-EVs recapitulate the therapeutic effects of the cells against acute GVHD [93]. A systemic infusion of MSC-EVs in mice with acute GVHD was associated with the suppression of CD4^+^ and CD8^+^ T cells and with the preservation of circulating naive T cells, possibly due to the unique microRNA profiles of MSC-EVs. The analysis on microRNA cargo in MSC-EVs identified that their target genes were involved in regulation of the cell cycle, T-cell receptor signaling, and GVHD [93]. These findings suggest that MSC-EVs could be a new potential therapeutic option to prevent GVHD, to be tested in future clinical trials.

## 10. Conclusions

The organ demand is continuously increasing and there is a constant need to expand the pool of donors. Increasing organ availability represents a major challenge in the field of transplantation.

Among the most recent innovative strategies, the use of EVs seems very promising. The application of EVs in the perfusion solution, recently proposed for kidney, lung, and liver grafts, results in the amelioration of donor organ viability and functionality. Moreover, consolidated results describe the beneficial effects of EV administration in several preclinical models of IRI. In particular, stem-cell-derived EVs have displayed strong pro-regenerative properties in different models of renal, lung, liver, and heart injuries. IRI is an unavoidable consequence after transplants and the severity of this phenomenon affects the graft outcome, leading to delayed graft function, graft rejection, chronic rejection, and chronic graft dysfunction. The development of strategies to limit the progression of IRI is fundamental for the success of transplants. Altogether, EVs appear the ideal candidate to target different aspects during transplantation process.

## Figures and Tables

**Table 1:** List of EV applications for organ preconditioning. Abbreviations: bone marrow (BM), human liver stem cells (HLSCs).

Organs	EV Sources	Type of Perfusion	Time of Preconditioning	Results	References
Kidney	BM-MSCs	Hypothermic	4 h	Preservation and protection	Gregorini et al. [58]
Lung	BM-MSCs	Normothermic	6 h	Improvement of ventilation and hemodynamic parameters	Gennai et al. [68]
Lung	BM-MSCs	Normothermic	6 h	Restoring permeability and reduction of inflammation	Park et al. [69]
Lung	BM-MSCs	Normothermic	1 h	Attenuation of IR dysfunction and immunomodulation	Stone et al. [70]
Lung	BM-MSCs	Normothermic	3 h	Reduction of inflammation and oxidative stress	Lonati et al. [71]
Liver	HLSCs	Normothermic	4 h	Limitation of the progression of ischemic injury	Rigo et al. [72]

**Table 2:** Immunomodulatory properties of MSC secretome/EVs.

Cell Types	Actions	Mechanisms	Effector	References
T lymphocytes	Decrease of TH1 secretion of IFN-γ [91] Increase of TH2 secretion of IL-4 [91] Increase of the proportion of T-regs [91] Suppression of T-naïve differentiation [94] Decrease in proliferation and migration [94] Decrease of CD4^+^CD8^+^ [94]	Constitutive production of COX2 and PGE2 [91,92,93] Secretion of TGF-β [91] Secretion of soluble HLA-G5 [93]	Secretome [91,92,93] EVs [94]	S. Aggarwal et al. [91] Z Selmani et al. [93] S. Fujii et al. [94]
DC	Reversion of maturation of DCs [92] Decrease DC1 production of TNF-α [91] Increase DC2 production of IL-10 [91]	Secretion of IL-6 [91]	Secretome [91,92]	S. Aggarwal et al. [91] F. Djouad et al. [92]
NK	Inhibition [91] Alteration of secreted cytokines [91]	Secretion of indoleamine 2,3-deoxygenase [91] Secretion of PGE2 [91] Secretion of TGF-β [91]	Secretome [91]	S. Aggarwal et al. [91]
